# Harmonization of GEOV2 fAPAR time series through MODIS data for global drought monitoring

**DOI:** 10.1016/j.jag.2019.03.017

**Published:** 2019-08

**Authors:** C. Cammalleri, A. Verger, R. Lacaze, J.V. Vogt

**Affiliations:** a European Commission, Joint Research Centre (JRC), Ispra, Italy; b CREAF, Cerdanyola del Vallès, 08193, Catalonia, Spain; c Hygeos, Earth Observation Department, Lille, France

**Keywords:** Temporal consistency, Anomalies, fAPAR, GEOV2, MODIS, Drought, EDO

## Abstract

The temporal consistency of the fAPAR GEOV2 full time series (constituted by data derived from SPOT-VGT1/2 and PROBA-V) is analyzed against the single-sensor MODIS dataset, with a particular focus on the most recent fAPAR anomalies (*z*-scores) produced from PROBA-V in the period 2014–2017. The intercomparison highlights a systematic overestimation of GEOV2 fAPAR *z*-scores when compared to MODIS fAPAR, likely related to the observed positive bias (over 90% of the domain) in the PROBA-V *vs.* SPOT-VGT1/2 relationship. A simple two-step harmonization procedure has been proposed to remove this discrepancy, based on two separate linear corrections of SPOT-VGT1/2 (2001–2013) and PROBA-V (2014–2017) data with respect to MODIS, followed by a time lag correction. The harmonized GEOV2 time series preserves the overall dynamic of fAPAR, while removing the sensor bias and improving the consistency with MODIS data. The fAPAR anomalies from the harmonized GEOV2 time series provide unbiased estimates of *z*-scores that are overall well correlated (*R* = 0.55 ± 0.25) with the MODIS fAPAR anomalies.

## Highlights

• A harmonization procedure of GEOV2 and MODIS fAPAR data is introduced.
• Systematic overestimations of GEOV2 anomalies compared to MODIS are removed.
• Temporal consistent standardized anomalies can be used in drought studies.

## 1 Introduction

The fraction of Absorbed Photosynthetically Active Radiation (fAPAR), defined as the fraction of solar radiation in the PAR spectrum absorbed by green elements of vegetation (Liang et al., 2012), plays a major role in both the water and the carbon budget of Earth’s land surface. The fAPAR is considered a good synthetic descriptor of the health status of the vegetation (Gobron et al., 2006), and its relevance in understanding land surface dynamics is highlighted by its inclusion in the United Nations Global Climate Observing System (GCOS) 50 essential climate variables (FAO, 2009).

Satellite remote sensing (RS) is considered one of the major sources of data for monitoring vegetation at regional to global scales, mainly due to its capability to cover a variety of spatial scales, the relative moderate cost for users, the extensive spatial coverage and high revisit frequency. The relevance of RS data in both natural vegetation and crop monitoring is confirmed by the large number of satellites dedicated to the observation of spectral quantities used to derived vegetation-related indicators (see e.g., Xie et al., 2008). Of particular interest for global applications are the so-called moderate resolution sensors (i.e., spatial resolution of about 250–1000 m) that provide a good compromise between spatial detail and temporal resolution; these include the currently operating MODIS (Moderate Resolution Imaging Spectroradiometer; https://modis.gsfc.nasa.gov), the PROBA-V (https://www.esa.int/Our_Activities/Observing_the_Earth/Proba-V) and the MetOp-AVHRR (Advanced Very High Resolution Radiometer on-board of the Meteorological Operational satellites; https://www.eumetsat.int/website/home/Satellites/CurrentSatellites/Metop/MetopDesign/AVHRR/index.html). Past operating sensors include the SPOT-VGT (Satellite Pour l’Observation de la Terre – VEGETATION, http://www.spot-vegetation.com), the MERIS (Medium Resolution Imaging Spectrometer, https://earth.esa.int/web/guest/missions/esa-operational-eo-missions/envisat/instruments/meris) and the SeaWIFS (Sea-Viewing Wide Field-of-View Sensor; https://eospso.nasa.gov/missions/sea-viewing-wide-field-view-sensor).

The data collected by these sensors have been used to estimate fAPAR dynamics by means of various retrieval algorithms (Baret et al., 2007; García-Haro et al., 2018; Gobron et al., 2006, 2007; Myneni et al., 2002; Verger et al., 2014) and validated under a wide range of land coverage and climatic conditions. Several studies have investigated the consistency between different retrievals (e.g., D’Odorico et al., 2014; Meroni et al., 2013; Pickett-Heaps et al., 2014), highlighting similar spatiotemporal patterns among different products but also major differences in the magnitude of the retrievals, particularly over forest areas.

Thanks to its sensitivity to vegetation water stress, fAPAR is a key variable in many environmental applications, including operational drought monitoring. The use of fAPAR in Drought Early Warning Systems (DEWS), and in particular of fAPAR deviations from the climatological historical conditions (i.e., anomalies or *z*-scores), has been investigated in a number of studies (Fensholt et al., 2004; Gobron et al., 2005; Ivits et al., 2016; Rossi et al., 2008). Most notably, fAPAR anomalies are currently used as one component in calculating both the Combined Drought Indicator (CDI) in the European Drought Observatory (EDO, http://edo.jrc.ec.europa.eu) (Sepulcre-Cantó et al., 2012), and the Risk of Drought Impact (RDrI) indicator in the Global Drought Observatory (GDO, http://edo.jrc.ec.europa.eu/gdo/).

Due to the need of reference baseline statistics for the computation of fAPAR anomalies, the temporal consistency in the time series is crucial in drought applications, especially over areas characterized by modest year-to-year fluctuations. Temporal inconsistencies may lead to systematically biased estimates of the anomalies and incorrect assessments of drought events. Since this strong sensitivity to temporal inconsistencies is especially relevant for drought studies, time series that can be considered temporally consistent for other applications may not be sufficiently accurate in this case.

In this framework, the availability of temporally consistent long-term fAPAR time series is an ideal condition that, unfortunately, cannot always be satisfied. Data collected by the same sensor on-board of the same satellite mitigate some of the inconsistency issues (even if sensor degradation and orbital drifting can alter the temporal consistency), whereas data from multiple instruments need to be accurately inter-calibrated to archive temporally consistent time series. Currently, the only single-sensor providing a reasonably long time series of data is the MODIS, which started collecting data in late 2000 on-board of the Terra satellite. This is the dataset currently used in EDO and GDO for a near-real time monitoring of fAPAR anomalies with 10-daily (dekadal) updates. However, this sensor is well beyond its designed life-time of 6 years, suggesting the need to identify alternative solutions for the upcoming future, also considering the sensor degradation observed in the most recent years (Lyapustin et al., 2014). Replacement sensors have already been launched, such as the VIIRS (Visible/Infrared Imager/Radiometer Suite) on-board of the S-NPP (Suomi National Polar-orbiting Partnership) satellite, with some studies already investigating the consistency between the two datasets (e.g., Skakun et al., 2018).

The COPERNICUS Global Land Service (https://land.copernicus.eu/) has faced the issue of data inconsistency since the PROBA-V sensor was launched in 2013 as successor of the SPOT series of VEGETATION instruments (VGT1 on SPOT-4 (launched in 1998) and VGT2 on SPOT-5 (launched in 2002)). The version 2 of the COPERNICUS 1-km fAPAR product (hereafter referred to as GEOV2) provides near-real time, continuous series of fAPAR maps starting in 1999 by combining data from SPOT-VGT1/2 and PROBA-V (Verger et al., 2017). This product has been extensively validated and cross-compared with previous versions, as well as with MODIS data, highlighting a complete spatial coverage, good temporal smoothness and spatio-temporal continuity (Camacho et al., 2018). On the one hand, the validation against ground data shows reasonable results (root mean square error ˜ 0.1), with a slight tendency of the PROBA-V product to overestimate fAPAR over the observation sites (bias ˜ 0.05) against a slight underestimation of SPOT-VGT1/2 estimates (˜ -0.04). On the other hand, Fuster et al. (2018) highlight some systematic discrepancies in the transition from SPOT-VGT2 to PROBA-V, which affect the capability to accurately capture the inter-annual variations. The impact of such inconsistencies on fAPAR anomalies, and consequent drought estimates, has not been fully quantified.

The goal of the current study is to evaluate in detail the temporal consistency of the GEOV2 fAPAR dataset, with a particular focus on dekadal fAPAR anomalies retrieved in the most recent years when PROBA-V data are used. A simple harmonization procedure of the GEOV2 dataset is here proposed in order to minimize the systematic inconsistencies (in terms of fAPAR anomalies) with the MODIS dataset currently used within EDO and GDO. This specific MODIS time series is used for the harmonization of GEOV2 *z*-scores not only because it is based on a single-sensor input data, which improves the temporal consistency of time series, but also due to its successful employment in operational drought monitoring.

## 2 Material and methods

In this section, both the GEOV2 and MODIS fAPAR datasets are introduced, including a brief description of the major spatio-temporal traits of the data. Additionally, the simple procedure adopted to minimize the internal discrepancies of GEOV2 in the transition between SPOT-VGT1/2 and PROBA-V by using MODIS data is described.

### 2.1 GEOV2 dataset

The GEOV2 fAPAR product is derived from SPOT-VGT data from 1999 until 2013 (combining data from SPOT4-VGT1 and SPOT5-VGT2) and successively from PROBA-V. GEOV2 fAPAR is computed, jointly with LAI (Leaf Area Index) and FCOVER (Fraction of vegetation coverage), from top of the canopy daily reflectances in the visible, near infrared and shortwave infrared bands, at 1-km resolution. Daily fAPAR values are retrieved using a neural network algorithm trained on fused MODIS and CYCLOPES products (Verger et al., 2014). A multi-step filtering, temporal smoothing, gap filling and compositing procedure is then applied to the daily estimates to generate the final dekadal products. Data are provided at the end of each dekad (10th, 20th and last day of the month; 36 images per year), with a temporal basis for compositing between 30 and 120 days depending on the number of available observations. A first estimate is delivered within 3 days in near-real time, which is successively updated within a consolidation period of six dekads (Verger et al., 2017).

Validation exercises based on the comparison with ground measurements on a limited dataset quantified the accuracy of the GEOV2 product to be about 0.1 fAPAR units, while also highlighting the marginal impact of the transition between SPOT-VGT1 and SPOT-VGT2 occurred in 2003 (Sánchez-Zapero and Camacho, 2017). For this reason, we are referring to this dataset as SPOT-VGT only from here onward. On the contrary, successive analyses highlighted an overall higher bias in PROBA-V estimates compared to SPOT-VGT (Fuster et al., 2018), with some inconsistencies in the transition from SPOT-VGT to PROBA-V also noticed, with a systematic positive bias of about 6% for PROBA-V.

Even though the same algorithm is adopted to process both SPOT-VGT and PROBA-V data, there are some differences between the two sensors that may result in the noted inconsistencies in the transition from SPOT-VGT to PROBA-V. For instance, the spectral bands are relatively close, but a slight shift in the spectral response function of the shortwave infrared bands exists (Dierckx et al., 2014). Other differences are related to the spatial sampling of the data and in the acquisition time. A series of pre-processing procedures are applied to minimize these discrepancies (including spectral linear correction of PROBA-V reflectances to mimic SPOT-VGT); still, these may represent sources of uncertainty in the consistency of the time series.

In this study, we used the consolidated GEOV2 product (the so-called RT6) for the period 2001–2017. The use of this consolidated dataset, rather than the RT0 available in near-real time, allows quantifying the temporal inconsistency without including also the uncertainty related to the near-real time estimates. This dataset includes 468 images from SPOT-VGT and 144 from PROBA-V covering the entire globe. Given the focus of the study on standardized deviations from the climatology, the cells where minimum inter-annual variability is observed (e.g., deserts and tropical evergreen broadleaf forests) were excluded from the analysis since in these cases standard deviation values are often close or equal to zero. The related areas were identified using the quality flag associated to the GEOV2 products, which includes the identification of bare soils and evergreen broadleaf forests, as well as the standard deviation (see Eq. (1) in Section 2.3). The latter was used to exclude areas with values < 0.01. Additionally, data with low accuracy (RMSE > 0.5 in the ancillary layers) were excluded as well. The data after the masking are re-sampled on a regular lat/lon grid at 0.01 ° resolution using the nearest neighbor method.

### 2.2 MODIS dataset

The MODIS fAPAR data used here as benchmark is the latest version standard MODIS product derived from the data collected by the sensor on-board of the Terra satellite (MOD15A2H, Collection 6) (Myneni, 2015). Both fAPAR and LAI maps are computed via a three-dimensional radiation transfer process through the canopy, starting from atmospherically corrected Bidirectional Reflectance Distribution Function (BRDF) values in seven spectral bands (Knyazikhin et al., 1998).

The Collection 6 of MOD15 aims at minimizing the effects of sensor degradation on the fAPAR and LAI produced maps. It should be noted that the MOD15 Collection 6 fAPAR product has only reached validation stage 1 (i.e., accuracy has been estimated using a small number of measurements, no evaluation was performed to estimate spatial/temporal consistency); and its overall accuracy is estimated at 0.15 fAPAR units RMSE, showing important overestimations over sparsely-vegetated areas (see also Yan et al., 2016). However, a robust estimation of anomalies favors temporal consistency over absolute accuracy of the estimates (see Section 2.3).

In the Collection 6, the fAPAR maps are generated daily at 500-m spatial resolution in a Sinusoidal projection, and successively delivered as 8-day composites using a maximum value compositing method. For the scope of EDO and GDO, the standard MODIS product is spatially aggregated at 1-km resolution and dekadal maps are derived. The spatial aggregation of the data is performed by a simple average of the 500-m data, and by successively re-projecting the aggregated data on a 0.01 ° lat/lon regular grid using the nearest neighbor method. The temporal aggregation at dekad scale is performed in two steps: i) calculation of the weighted average of the two closest 8-day images (weights equal to the number of overlapping days with the dekad), and ii) exponential smoothing of the dekad data (with smoothing parameter equal to 0.5; Brown and Meyer, 1961), which averages the previous dekad smoothed fAPAR and the current raw data in order to reduce the impact of abrupt changes in fAPAR time series due to misestimates. Both aggregation procedures are performed only on the data marked as good quality (based on the first bit of the product QA flag). The dataset used here includes all the MOD15A2H tiles (about 290 for each date) available for the period 2001–2017, which includes a total of 782 8-day composite dates.

### 2.3 fAPAR anomalies

The analysis focuses on fAPAR standardized anomalies, *z*, defined as:

(1) $$\begin{array}{ccc} z_{i,j}=\frac{\text{fAPA}{\text{R}}_{i,j}-\mu_{i}}{\sigma_{i}} & \text{with} & \begin{array}{c} \mu_{i}=\frac{1}{N}\sum_{j=1}^{N}\text{fAPA}{\text{R}}_{i,j}\\ \sigma_{i}=\sqrt{\frac{\sum_{j=1}^{N}{\left(\text{fAPA}{\text{R}}_{i,j}-\mu_{i}\right)}^{2}}{N}} \end{array} \end{array}$$

where the subscripts *i* and *j* represent the dekad (1–36) and the year, respectively, and *N* is the total number of years in the baseline dataset. This relationship is applied independently for each grid cell of the domain, but the spatial subscripts are omitted for the sake of simplicity.

Commonly, in drought analysis a value of |z| = 1 is often used to discriminate possible wet (positive anomalies, z > 1) or dry (negative anomalies, z < -1) conditions from the normal ones (e.g., see McKee et al., 1993). On a global average and for a relatively long time-span, the occurrence of *z*-scores within these three ranges is expected to resemble the one from a normal distribution (about 16% for each tail and 68% for the central portion of the distribution).

### 2.4 Consistency assessment

Temporal consistency of the GEOV2 dekadal *z* time series (using baseline statistics computed on the full 2001–2017 period) is evaluated by comparison with the analogous MODIS dataset. The consistency between the two datasets is evaluated by means of the Mean Absolute Difference (MAD) and BIAS metrics, defined as:

(2) $$MAD=mean\left(\left|z_{MOD}-z_{GEOV2}\right|\right)$$

(3) $$BIAS=mean\left(z_{MOD}-z_{GEOV2}\right)$$

Considering the use of |z| = 1 as discriminator for extreme conditions, in this analysis we assumed, as a rule of thumb, a value of MAD = 0.5 (i.e., half of a standard deviation) as the upper limit to discriminate between acceptable and unacceptable discrepancies.

Additionally, the temporal consistency in fAPAR time series was assessed by comparing the baseline statistics computed on different periods: (i) the full 2001–2017 period, and (ii) the SPOT-VGT period 2001–2013, avoiding possible inconsistency in the transition between SPOT-VGT and PROBA-V data (occurred at the beginning of 2014). These two periods were chosen in order to have enough data (> 10 years) to compute robust baseline datasets (contrarily to the PROBA-V 2014–2017 period). The two sets of baseline statistics (see Eq. (1)) were compared in terms of BIAS.

### 2.5 Harmonization procedure

The adopted procedure to harmonize the GEOV2 dataset with respect to MODIS is based on a lagged linear transformation to match the average and standard deviation of the time series of the two datasets as:

(4) $$\begin{array}{ccc} \text{fAPA}{{\text{R}}^{\prime }}_{2,k}=\text{fAPA}{\text{R}}_{2,k-d}A+B & \text{with} & \begin{array}{c} A=\frac{S_{1}}{S_{2}}\\ B=M_{1}-M_{2}\frac{S_{1}}{S_{2}} \end{array} \end{array}$$

where the prime superscript denotes the harmonized time series, *k* is the time-step (between 1 and 36×*N*), the subscripts 1 and 2 represent MODIS and SPOT-VGT/PROBA-V data, respectively, *M* and *S* are the full dataset long-term average and standard deviation (which differ from the dekadal values in Eq. (1)), and the *d* parameter represents the temporal lag. All three harmonization parameters are computed independently for each cell, with *A* and *B* being independent from the estimation of *d*.

This approach allows preserving the year-to-year fluctuations by removing only the systematic discrepancies between SPOT-VGT and PROBA-V anomalies. This is obtained by applying the harmonization procedure separately for SPOT-VGT (2001–2013) and PROBA-V (2014–2017) datasets, forcing both time series to be consistent to MODIS, and, therefore, also consistent to each other. The introduction of a lag parameter aims at taking into account a possible temporal shift between MODIS and GEOV2 time series, likely related to the different time-aggregation procedures adopted by the two datasets (see Sections 2.1 and 2.2).

In detail, the harmonization procedure is applied in a two-step approach; in the first step, the parameters *A* and *B* are computed separately for SPOT-VGT and PROBA-V datasets against their corresponding MODIS dataset (2001–2013 and 2014–2017, respectively). Successively, in the second step, the scaled time series are compared against the full MODIS dataset (2001–2017) in a set of lagged correlation analyses performed with seven different time lags (between ‒3 and +3). The “optimal” lag (*d*), used to correct the data via Eq. (4), is then defined as the one corresponding to the highest statistically-significant Pearson correlation coefficient, *R*, defined as:

(5) $$R=\frac{\sum_{k=1}^{N}\left(fAPAR_{1,k}-M_{1}\right)\left(fAPAR_{2,k-d}-M_{2}\right)}{\sqrt{\sum_{k=1}^{N}{\left(fAPAR_{1,k}-M_{1}\right)}^{2}}\sqrt{\sum_{k=1}^{N}{\left(fAPAR_{2,k-d}-M_{2}\right)}^{2}}}$$

The statistical significance of the *R* values is tested by comparison with the *R* value corresponding to a significance level, *p*, of 0.05 according to the t-Student test (2 sided). Additionally, the statistical significance of the difference between the *R* value corresponding to the “optimal” lag and the one for *d* = 0 is evaluated by applying the Fisher transformation to the *R* values, and then by computing the *p*-value (2 sided) associated to the deviation of the standard error from zero (see Steiger, 1980).

**Fig. 1:**
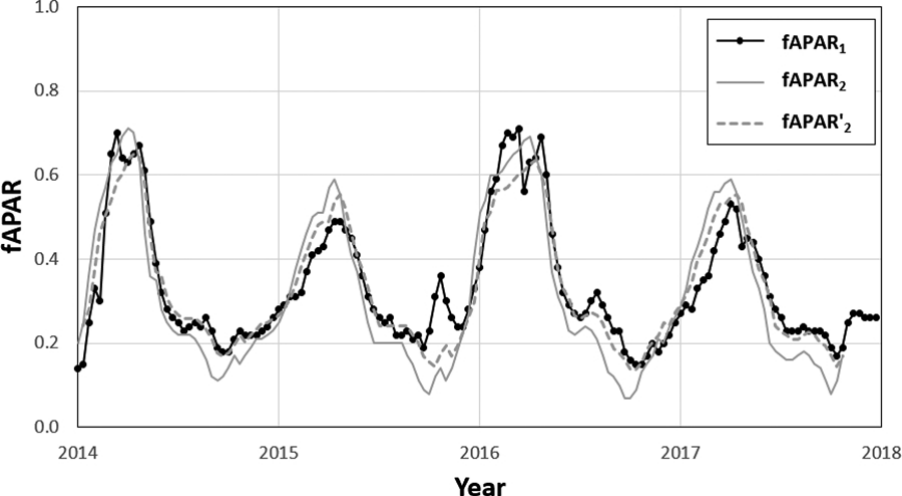
Example of the harmonization procedure applied to PROBA-V data collected between 2014 and 2017 in Tunisia (9.91E, 35.56 N). fAPAR1 refers to MODIS time series, fAPAR2 to the original PROBA-V time series and fAPAR’2 to the harmonized PROBA-V time series. Parameters in this case are: *A* = 0.78, *B* = 0.08 and *d* = 1.

The two-step harmonization procedure allows accounting for the likely different bias and variability of the two GEOV2 sub-datasets compared to MODIS, while also keeping the temporal correspondence among dekadal values of the two original sub-datasets (since the same lag is applied to the full GEOV2 time series). In summary, the full harmonization procedure requires the computation of two pairs of *A* and *B* maps (one for SPOT-VGT and one for PROBA-V) and a single *d* map. An example of this harmonization procedure for a single cell is depicted in Fig. 1, where actual PROBA-V data collected over an agricultural area in Tunisia for the period 2014–2017 are processed to match, on average, the dynamic of MODIS data in the same period. This example clarifies how the general dynamic of the original data remain intact, while the systematic differences are removed.

## 3 Results and discussion

### 3.1 Consistency between GEOV2 and MODIS anomalies

As a preliminary test, the fAPAR anomalies derived from the PROBA-V data for the period 2014–2017, based on GEOV2 baseline statistics computed on the full 2001–2017 period (hence, mixing SPOT-VGT and PROBA-V data), were compared against the MODIS-derived anomalies for the same years (and the same baseline period). The focus on the most recent years only is dictated by the final goal to use those products for a near-real time monitoring of drought.

**Fig. 2:**
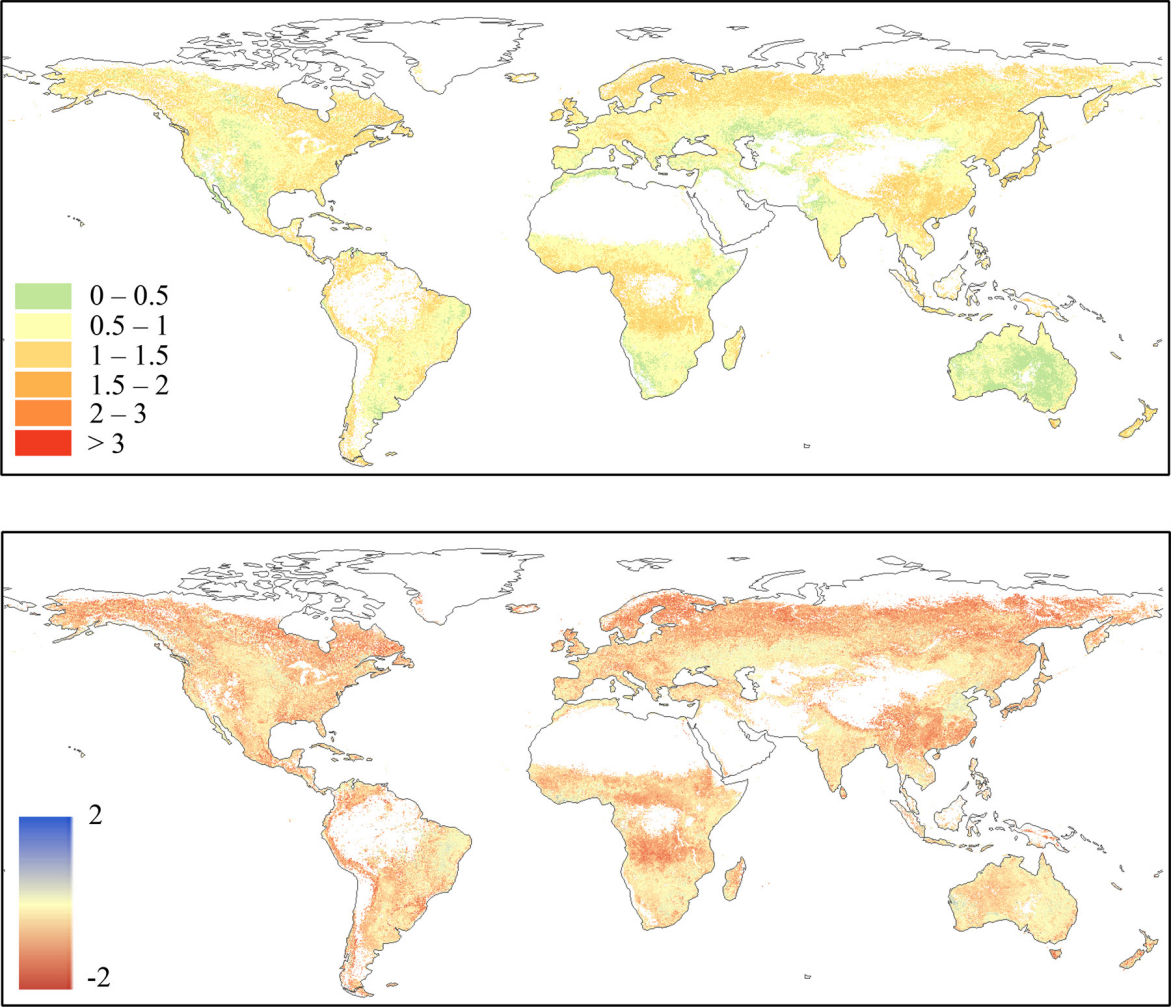
Spatial distribution of MAD (upper panel) and BIAS (lower panel) statistics computed on the period 2014–2017 by comparing MODIS and GEOV2 *z*-scores. Negative BIAS values represent an overestimation of GEOV2.

The spatial distribution of MAD and BIAS statistical indices is reported in Fig. 2, where areas with more than 1/3 of the dekad *z*-scores missing in either GEOV2 or MODIS are masked out. These missing values are mainly due to winter cloud coverage and low solar angle (mostly over northern latitude), as well as the already discussed masked areas over tropical forests and desert areas.

The MAD map in Fig. 2 (upper panel) highlights a good consistency of the two datasets over Australia, central US, parts of Argentina and Brazil, south-western Africa, the Horn of Africa, the Central Asia, and Pakistan, whereas large MAD values (> 1) are observed over central Africa, south and central China and the northern latitudes. The BIAS map (Fig. 2, lower panel) shows mostly negative values (overestimation of GEOV2 anomalies compared to MODIS ones), with very few notable exceptions (i.e., northeast Brazil, northeast China, and the West African coast).

**Fig. 3:**
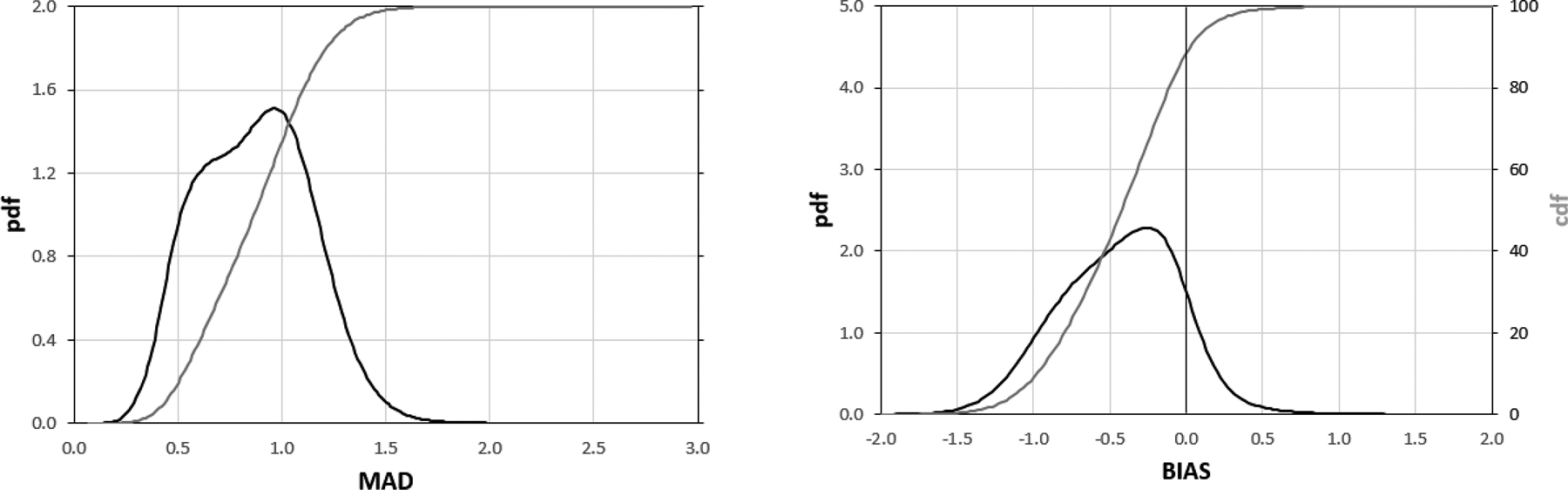
Frequency distribution of the MAD (left panel) and BIAS (right panel) data reported in Fig. 2. The *pdf*s are represented by the black lines (on the left y-axes), whereas the *cdf*s are represented by the grey lines (on the right y-axes). (For interpretation of the references to colour in this figure legend, the reader is referred to the web version of this article).

The plots in Fig. 3 summarize the frequency distribution of the MAD (left panel) and BIAS (right panel) values obtained from this analysis. These plots allow easily evaluating the fraction of the globe under a specific MAD or BIAS value, as well as the most frequent values for both statistics.

The *cdf* (cumulative distribution function) of the MAD values (Fig. 3, left panel) highlights that only a small fraction of cells has MAD < 0.5 (about 10%), while about 35% of the cells have MAD > 1. Overall, most of the data are within the range 0.5–1, with a median MAD equal to 0.86 and an interquartile range of 0.4. The analysis of the BIAS *cdf* (Fig. 3, right panel) shows how most of the cells (about 90%) have a negative bias (GEOV2 overestimates MODIS *z* values), a result also highlighted by the mode and the median equal to about -0.2 and -0.42, respectively, and the practical absence of blue-shaded cells in Fig. 2.

### 3.2 Impact of PROBA-V data on baseline statistics

**Fig. 4:**
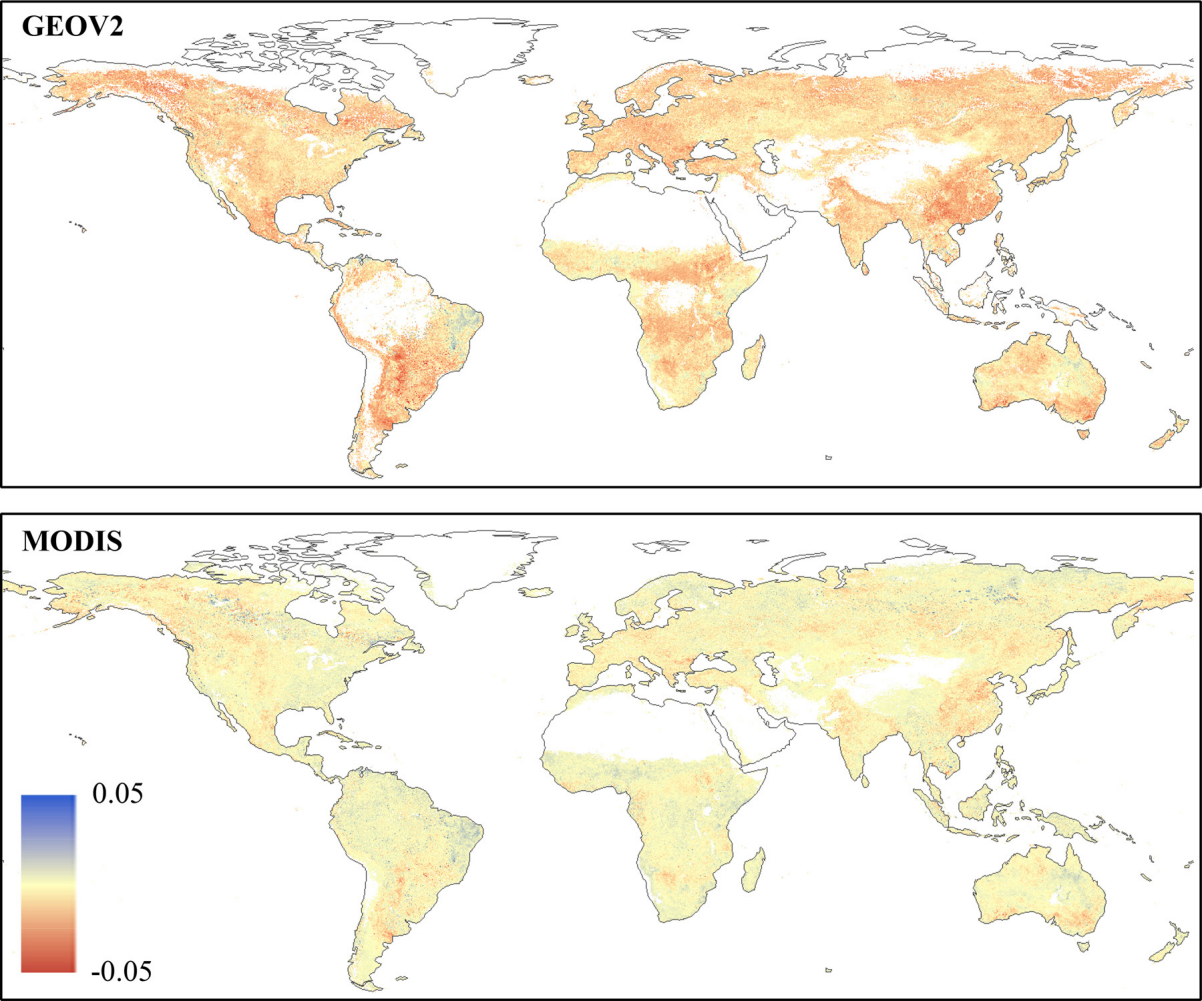
Spatial distribution of the BIAS statistic computed by comparing the dekadal average baseline values (see Eq. (1)) on the period 2001–2013 (SPOT-VGT period) and 2001–2017 (full time series) for GEOV2 (upper panel) and for MODIS (lower panel) fAPAR time series. Negative BIAS values represent an overestimation of the baseline values on the period 2001–2017 compared to 2001–2013.

**Fig. 5:**
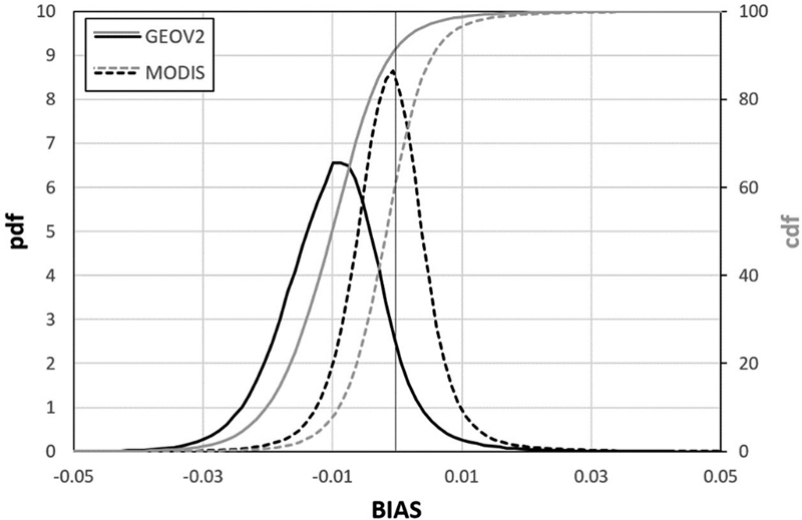
Frequency distribution of the BIAS data reported in Fig. 4. The *pdf* is represented by the black line (on the left y-axes), whereas the *cdf* is represented by the grey line (on the right y-axes). Continuous lines refer to GEOV2 data, whereas dotted lines represent the MODIS data. (For interpretation of the references to colour in this figure legend, the reader is referred to the web version of this article).

This systematic slight overestimation of PROBA-V *z*-scores compared to MODIS observed in section 3.1 may be related to an overestimation of PROBA-V fAPAR values compared to SPOT-VGT. In order to corroborate this hypothesis, the two baselines computed for the period 2001–2017 (including both SPOT-VGT and PROBA-V data) and for the period 2001–2013 (only SPOT-VGT data) were compared. The map in Fig. 4 (upper panel) shows the spatial distribution of the BIAS between the dekadal average values (*μ*_i_ in Eq. (1)) of the two baselines (SPOT-VGT only ‒ full GEOV2), along with the BIAS calculated between the two MODIS baselines computed on the analogues time periods (lower panel). Additionally, the plot in Fig. 5 reports the corresponding frequency and cumulative distributions for both GEOV2 and MODIS.

The map in Fig. 4 (upper panel) shows a predominance of negative values (2001–2013 underestimates 2001–2017), with few notable exceptions over the same areas highlighted for Fig. 2 (lower panel), with which some clear similarities in the spatial patterns are noticeable, even if not a perfect 1 to 1 correspondence. The analogous map for MODIS (lower panel) highlights a higher concentration of values close to zero, with some similar patterns in correspondence of the areas with more extreme values (e.g., eastern China and central Latin America). Cammalleri and Vogt (2018) show that about 10% of the globe has significant fAPAR trend in MODIS data, mostly constituted by increasing values, a result that is spatial consistent with the areas with large differences in Fig. 4 (lower panel).

The GEOV2 *cdf* in Fig. 5 (continuous grey line) clearly shows the unusual high fraction of negative values (about 90%), with a peak of the *pdf* (probability density function, continuous black line) centered at about -0.01. It is worth mentioning how in a consistent dataset a symmetric distribution centered on zero is expected, similarly to what is observed for the MODIS dataset (dotted lines). Overall, these plots confirm a limited impact of the trends observed in Cammalleri and Vogt (2018) on the baseline statistics.

The overestimation of 2001–2017 average baseline compared to the 2001–2013 one seems to confirm a systematic overestimation of PROBA-V data compared to SPOT-VGT, which can be the cause of the overestimation of fAPAR anomalies compared to MODIS. This result also highlights how the use of a SPOT-VGT only baseline to compute PROBA-V *z* values would drive to even further biased estimates. It is worth highlighting how the BIAS observed for the average baseline values is relatively small on average, hence trivial for studies focusing on the fAPAR values itself. However, they are comparable with the climatological year-to-year variations (i.e., standard deviation of the baseline, not shown).

Overall, the results reported in this section suggest a tendency of PROBA-V data to slightly overestimate the SPOT-VGT ones over a large fraction of the globe (about 90%), causing a systematic bias between GEOV2 and MODIS fAPAR dekadal anomalies.

### 3.3 Analysis of the harmonization procedure

As described in Section 2.3, in the two-step harmonization procedure firstly the two pairs of *A* and *B* parameters are derived for SPOT-VGT and PROBA-V, separately, and used to scale the two datasets; hence, the lag parameter is computed on the scaled full dataset. The harmonized time series fAPAR^’^ is used to compute the corresponding baseline statistics and finally the anomalies for the period 2014–2017.

**Fig. 6:**
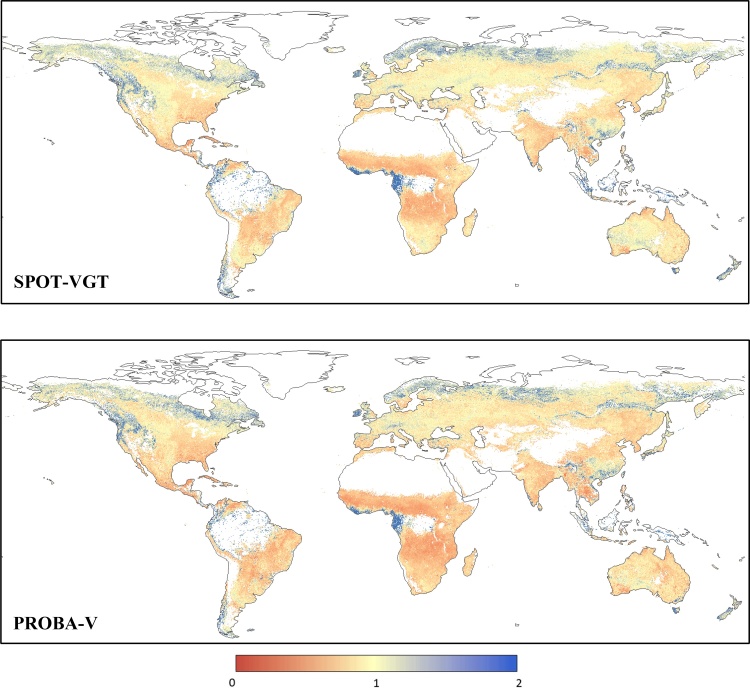
Spatial distribution of the *A* parameter (see Eq. (4) for details) for SPOT-VGT (upper panel) and PROBA-V (lower panel).

**Fig. 7:**
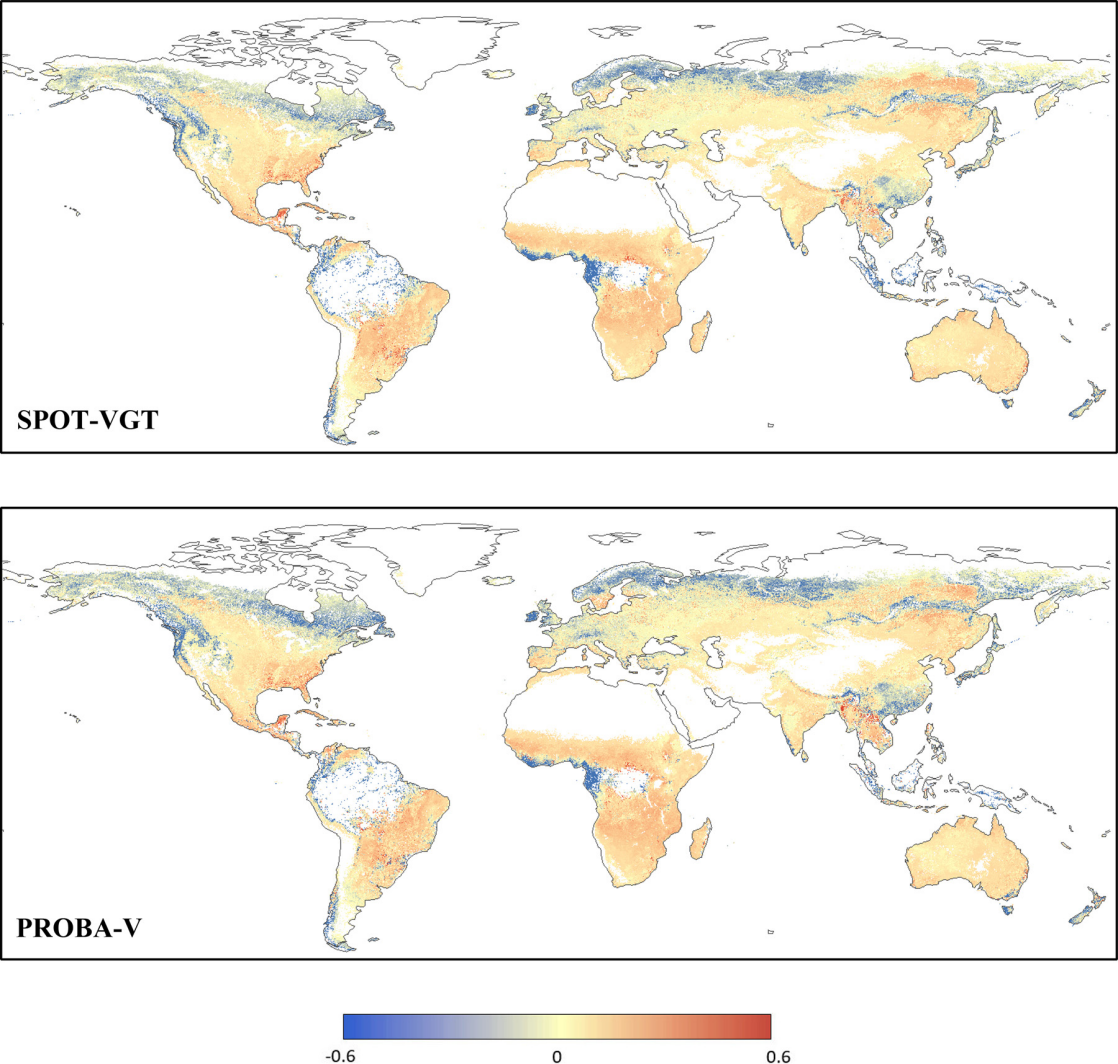
Spatial distribution of the *B* parameter (see Eq. (4) for details) for SPOT-VGT (upper panel) and PROBA-V (lower panel).

The maps in Fig. 6 depict the spatial distribution of the *A* parameters for the two GEOV2 sub-datasets, with the SPOT-VGT in the upper panel and the PROBA-V in the lower panel. Analogously, the maps in Fig. 7 report the *B* parameters for the SPOT-VGT (upper panel) and PROBA-V (lower panel).

The two maps of the *A* parameter in Fig. 6 (with mostly values < 1) show the tendency of both PROBA-V and SPOT-VGT to have a higher variability compared to MODIS (*S*_2_ > *S*_1_, see Eq. 4). Additionally, *A* is higher for SPOT-VGT compared to PROBA-V, particularly over southern Africa and eastern Asia. Given the role of *A* in Eq. (4), these results suggest larger annual variations in PROBA-V data compared to SPOT-VGT, with the latter closer to MODIS in its dynamic.

The two maps of *B* reported in Fig. 7 are relatively close in both spatial patterns and magnitude, with differences that are less marked than the ones observed in Fig. 6. Over few areas (e.g., India and north-eastern China) it is possible to notice slightly higher values in PROBA-V compared to SPOT-VGT. The similarity in these patterns with the ones observed in Fig. 6 is partially related to the fact that *B* is computed as the difference between two average values, one of which is scaled according to *A* (see definition of *B* in Eq. (4)).

Most of the cells for both PROBA-V and SPOT-VGT datasets display *A* < 1 and *B* > 0, and the areas with *A*>1 and *B*<0 values are mostly located in the equatorial belt and northern latitudes (i.e., Gulf of Guinea and Scandinavian Peninsula). This different behavior, however, is not going to significantly affect the homogenization procedure since both parameters are similar for the two datasets.

**Fig. 8:**
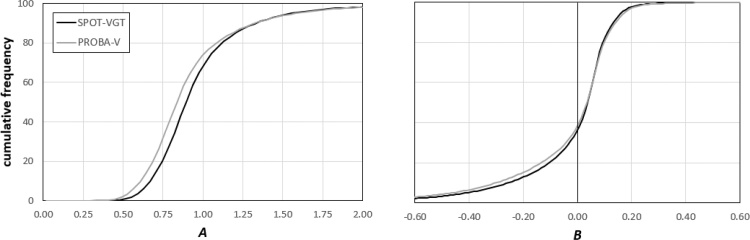
Cumulative frequency distribution of the *A* (left panel) and *B* (right panel) values obtained by constraining the SPOT-VGT (black lines) and the PROBA-V (grey lines) datasets to MODIS (For interpretation of the references to colour in this figure legend, the reader is referred to the web version of this article).

The general considerations extrapolated from a qualitative analysis of the maps in Fig. 6, Fig. 7 are further corroborated by the plots in Fig. 8, where the *cdf* of *A* values obtained for PROBA-V shows its underestimation compared to SPOT-VGT, whereas very similar behaviors are observed in the two *B* parameter *cdf*s. Overall, these results confirm the small but systematic differences between the average behavior of the two datasets compared to MODIS, with most of the differences related to differences in variability captured by the *A* parameter.

**Fig. 9:**
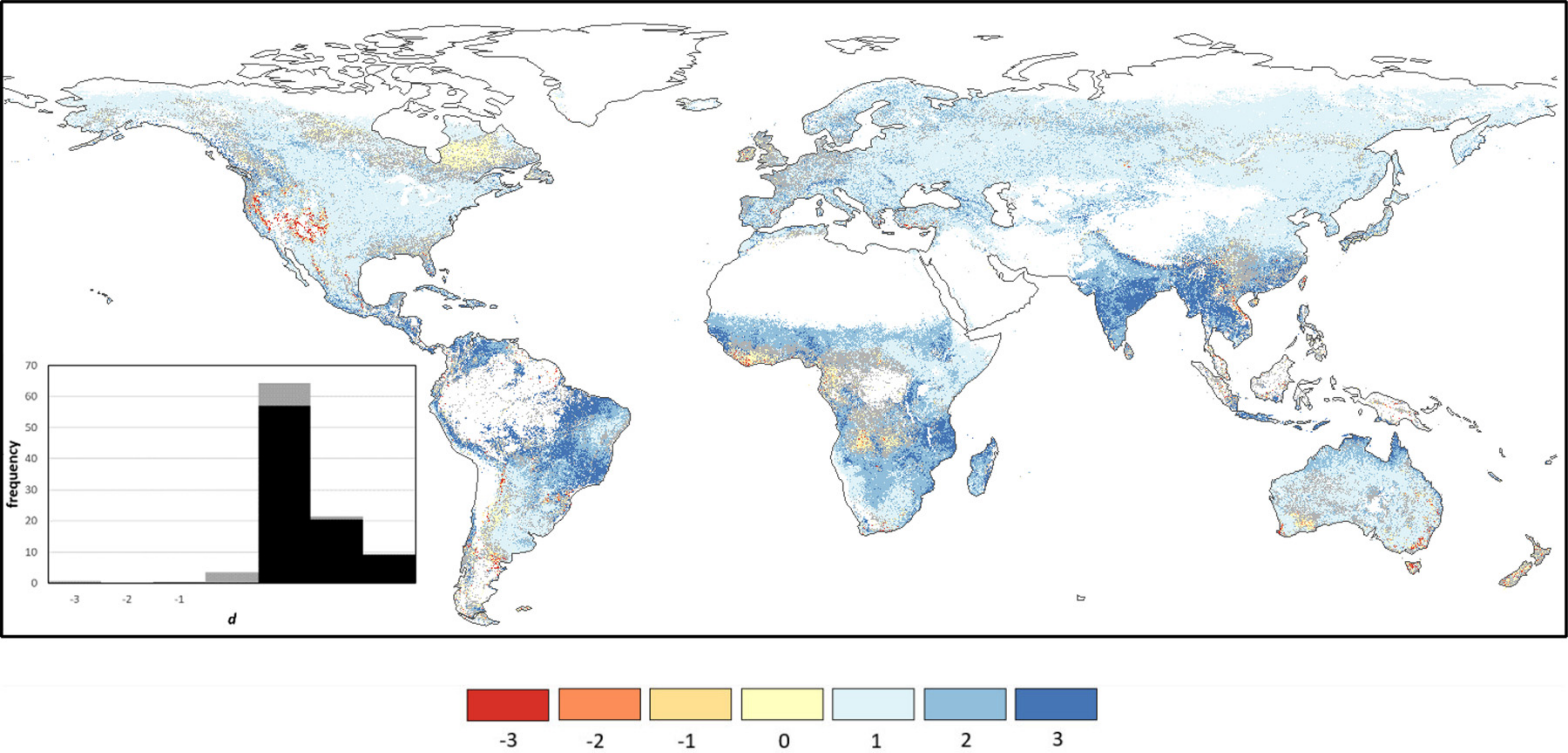
Spatial distribution of the optimal time lag between GEOV2 and MODIS full datasets according to the maximum Pearson correlation coefficient. Areas in grey are the ones with no statistically significant (*p* =  0.05) difference between the correlation coefficient for optimal lag and no-lag. The insert shows the frequency distribution of the obtained *d* values, with the portions in grey highlighting the fractions with no statistically significant improvements over the no-lag. (For interpretation of the references to colour in this figure legend, the reader is referred to the web version of this article).

Once the parameters *A* and *B* are computed, a lagged correlation analysis (on seven lag values) is performed between GEOV2 and MODIS in order to identify the optimal time lag for each cell (as a multiple of a dekad). The outcome of this analysis is reported in Fig. 9, where the spatial distribution of the *d* values is depicted (main map), as well as the frequency distribution of the values (insert). The cells with non-significant improvement in correlation (at *p* =  0.05) including a lag factor (compared to the no-lag hypothesis) are depicted in grey in both plots, whereas a value of *d* = 0 is imposed for the cells with non-significant correlation according to the t-Student test (at *p* =  0.05). In addition, the cells where more than 1/3 of the dekadal *z*-scores are missing were masked out.

The map in Fig. 9 shows some clear spatial patterns in *d* values, with high values over eastern Brazil, India, south-east Asia and some regions of southern Africa, whereas few negative values are observed over western USA and the southern coast of Australia. The frequency distribution of the *d* values (see insert in Fig. 9) highlights how a large percentage of the cells has a single positive (GEOV2 precedes MODIS) dekadal shift (65%), and a very small fraction (only about 2%) has negative (GEOV2 follows MODIS) *d* values. Overall, about 88.6% of the cells have a statistically significant increase in *R* due to the inclusion of a lag factor, with almost the entirety of data with non-significant improvement having *d* = 1. Considering the high fraction of the domain showing a statistically significant lag with *d* = 1 (about 57%), we decided to maintain the lag also for those cells where the improvement in *R* is observed but not statistically significant (about 8% of the cells).

The observed lags are likely related to the differences in the time-aggregation procedures adopted by the two datasets, since GEOV2 includes up-to 120 days in the aggregation (a value that may vary spatially depending on the number of observations available and that includes also frontward data), whereas MODIS combines only the two closest 8-day images (see sub-sections 2.1 and 2.2). In this regard, the positive values observed in the majority of the cells seem reasonable.

Given that negative *d* values correspond to an optimal correlation between “future” GEOV2 and “current” MODIS data (GEOV2 follows MODIS), and accounting for the limited percentage of data with *d* < 1 (about 2% of the cells), we adopted *d* = 0 for this small fraction of cells, whereas we used the optimal lag for all the other cells with *d* ≥ 0. This solution allows for a potential joint use of GEOV2 and MODIS in an early warning system without sacrificing the near-real time nature of the system. It is worth highlighting that this step is relevant only if the simultaneous use of GEOV2’ and MODIS datasets is foreseen; in all the other circumstances, the GEOV2’ dataset can be used without altering the temporal lag (i.e., *d* = 0) since there is no evidence of a better temporal dynamic of MODIS compared to GEOV2.

**Fig. 10:**
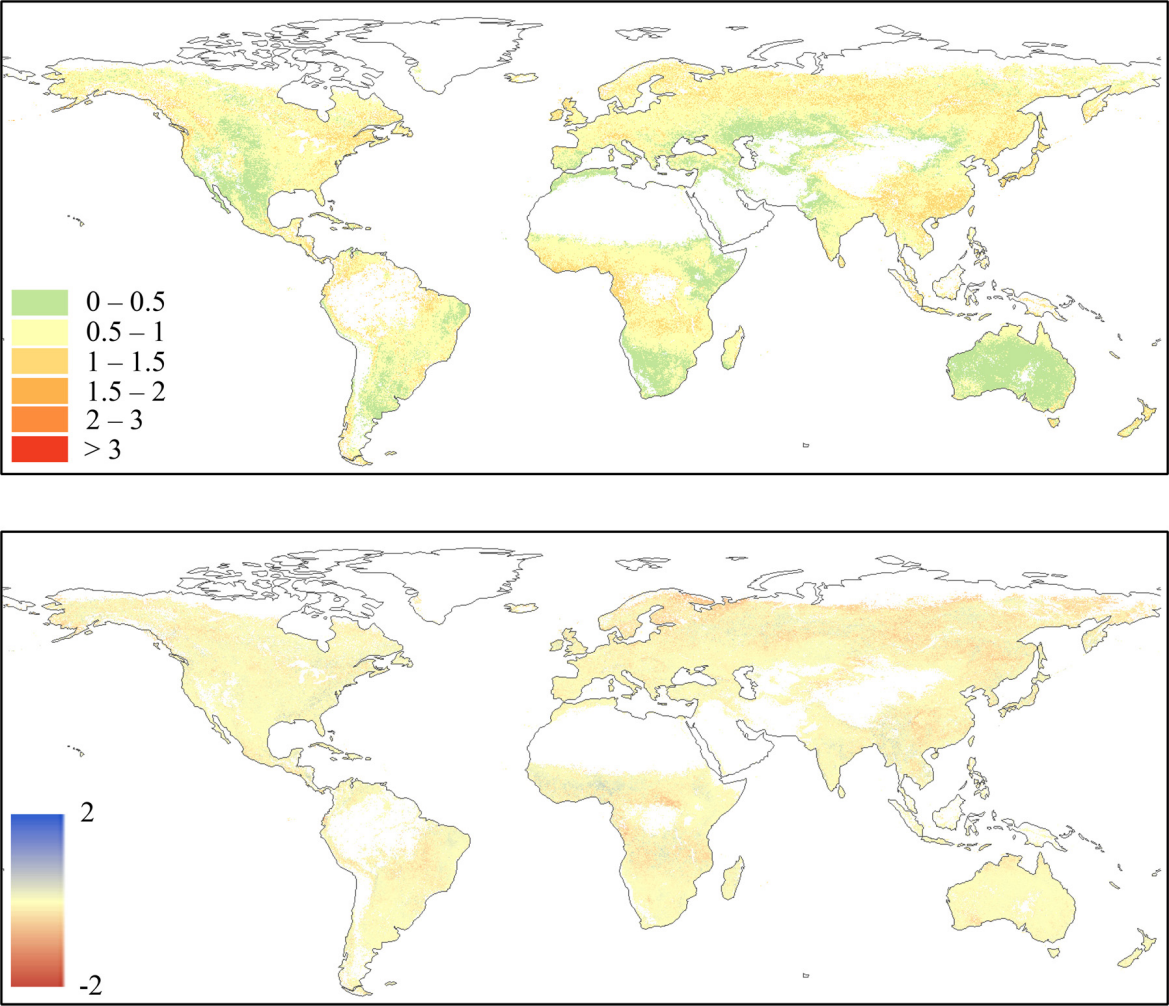
Spatial distribution of MAD (upper panel) and BIAS (lower panel) statistics computed on the period 2014–2017 by comparing MODIS and GEOV2^’^*z*-scores.

The maps in Fig. 10 show the MAD and BIAS statistics computed on the period 2014–2017 between fAPAR anomalies derived from MODIS and the corrected GEOV2 datasets (GEOV2’). These maps are analogous to the ones reported in Fig. 2, hence highlighting the impact of the correction procedure on the intercomparison.

**Fig. 11:**
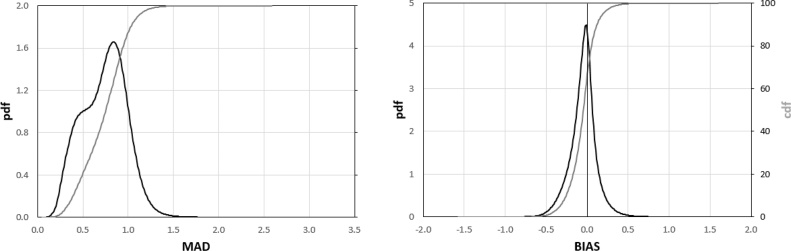
Frequency distribution of the MAD (left panel) and BIAS (right panel) data reported in Fig. 10. The *pdf*s are represented by the black lines (on the left y-axes), whereas the *cdf*s are represented by the grey lines (on the right y-axes) (For interpretation of the references to colour in this figure legend, the reader is referred to the web version of this article).

As it can be inferred by a visual comparison of the maps in Fig. 2, Fig. 10 (upper panels), the harmonized dataset shows smaller differences to the original GEOV2 over large areas (such as Australia, central US and southern Africa), as well as negligible BIAS over most of the globe (see Fig. 10, lower panel). The MAD and BIAS frequency distribution (Fig. 11) further corroborate these findings, showing how about 25% of the domain has MAD < 0.5 (*vs.* 10% for the original data) and how only 10% of the cells has MAD > 1 (*vs.* 35%). The MAD median and interquartile ranges are equal to 0.72 and 0.35, respectively, both showing a reduction from the values for the original GEOV2 dataset.

Additionally, the BIAS *pdf* is almost perfectly centered on the zero value (median equal to -0.04 vs. -0.42 for the original dataset) with two symmetric short-tails. Overall, the improved agreement between the harmonized data and MODIS compared to the original GEOV2 is evident, while at the same time the original dynamic of the latter is mostly preserved due to the simple linear bias correction adopted for SPOT-VGT and PROBA-V.

**Fig. 12:**
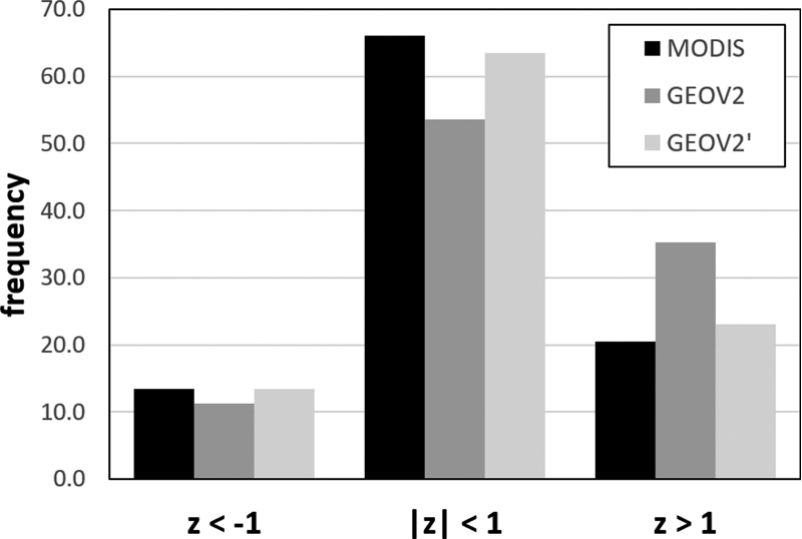
Average frequency of *z*-scores at global scale for the period 2014–2017.

Another test, specifically focused on extreme events, is summarized in Fig. 12, where the global average frequency of positive (*z* > 1) and negative (*z* < ‒1) extremes, as well as “normal” conditions (|*z*| ≤ 1), are quantified for the full period 2014–2017. As detailed in Section 2.3, these average frequencies are expected to resemble the normal distribution.

The data reported in Fig. 12 show how the harmonized GEOV2 dataset (GEOV2^’^) performs much closer to MODIS compared to the original GEOV2, with the latter characterized by an unusually high fraction of positive anomalies (above 35%, compared to the 20% of the other two datasets). Also in this case, the results are in line with the previously observed overestimation of anomalies by PROBA-V data. Overall, both MODIS and GEOV2^’^ seem to perform relatively close to the expected normal distribution and better than the original GEOV2. The higher frequency of positive anomalies compared to negative ones (about 20% *vs.* 13%) can be partially explained by the slight linear trend in MODIS data observed by Cammalleri and Vogt (2018) over some areas of the globe, which is also partially visible in the baseline differences reported in Fig. 4 (lower panel).

**Fig. 13:**
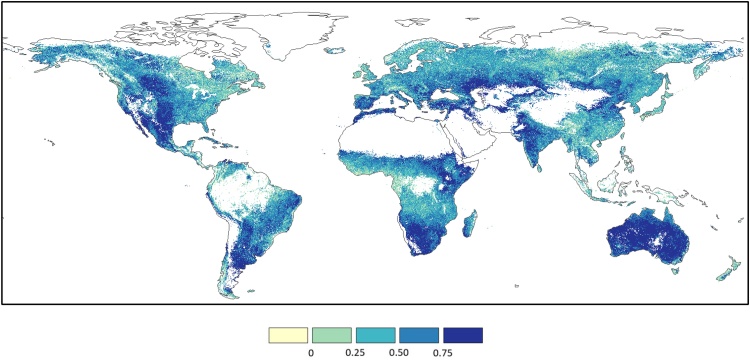
Spatial distribution of the *R* statistic computed between MODIS and GEOV2^’^ fAPAR anomalies on the period 2014–2017.

A synthetic representation of the agreement between the MODIS and the GEOV2^’^ *z* time series over the period 2014–2017 is depicted through the *R* coefficient map reported in Fig. 13. This map shows a very high correspondence between the two datasets over several regions, including most of the southern hemisphere, central US, India and the Mediterranean area. Overall, the *R* values result positive over most of the globe (> 90%), with only few sparse notable exceptions. The domain average *R* (0.55 ± 0.25) suggests a substantial agreement between the two datasets after the harmonization. As a benchmark, the *R* values for the original GEOV2 (not shown) have an average of 0.41 ± 0.29.

**Fig. 14:**
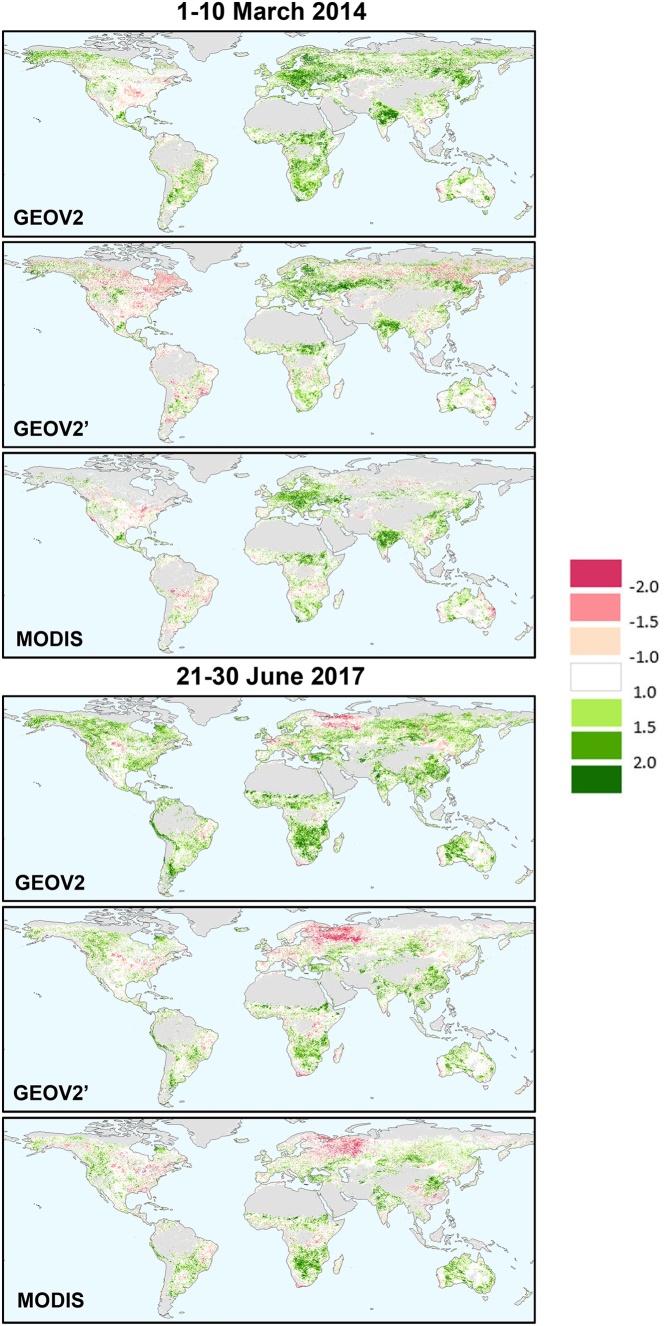
Examples of fAPAR *z*-scores for two dakads (first of March 2014 and last of June 2017) as obtained for the three datasets: GEOV2, GEOV2’ and MODIS.

Finally, two examples of the spatial distribution of *z* values for the three datasets (GEOV2, GEOV2’ and MODIS) are reported in Fig. 14, specifically for the first dekad of March 2014 and the last dekad of June 2017 (roughly at the end of winter and at the start of summer in the Northern hemisphere). These maps clearly show the similarities in the spatial patterns between MODIS and GEOV2’, whereas the original GEOV2 data show an abundance of positive anomalies, due to the positive bias in PROBA-V data, which is an unrealistic outcome of an anomaly analysis.

## 4 Summary and conclusions

Temporal consistency in fAPAR time series is a crucial characteristic when these data are used in Drought Early Warning Systems (DEWS), since DEWS usually rely on deviations from long-term climatological conditions (i.e., anomalies) rather than on absolute values. In this framework, small internal temporal inconsistencies in a time series that are commonly considered acceptable for other applications, may lead to significantly biased anomaly estimations that can result in an unreliable mapping of drought events.

The GEOV2 dataset, produced within the COPERNICUS Global Land Service, appears as a valuable source of global fAPAR data for use in DEWS, given its near-real time availability and accurate estimates. This product represents a potential alternative to the MODIS dataset, which is currently the only long-term fAPAR dataset available and acquired by a single satellite sensor. Unfortunately, the use of two different sensors in GEOV2 for the periods 2001–2013 (SPOT-VGT) and 2014-onward (PROBA-V) requires a cautious evaluation on the likely presence of an inconsistency between the two datasets.

The analysis of dekadal (roughly 10-day) fAPAR anomalies reported in this study, focusing on the period 2014–2017, highlights a clear bias between GEOV2 and MODIS, likely related to the slight overestimation of PROBA-V fAPAR data compared to SPOT-VGT. Since the baseline statistics used for the computation of GEOV2 fAPAR anomalies are largely grounded on SPOT-VGT data (13 years out of 17), the systematic discrepancy between PROBA-V and SPOT-VGT directly influences the accuracy of fAPAR anomaly estimates.

The simple harmonization procedure proposed here, which suggests forcing both SPOT-VGT and PROBA-V on the corresponding MODIS data, is an operational alternative to improve the usability of GEOV2 data in DEWS. Additionally, the lagged correlation analyses, adopted to remove a possible temporal lag between MODIS and GEOV2 time series, further constrain the consistency between the two datasets. This latter step is not necessary if GEOV2 and MODIS datasets are not used in conjunction. Overall, the GEOV2 dataset obtained after the harmonization (GEOV2^’^) is characterized by the absence of systematic biases when compared to MODIS, and by overall lower mean absolute differences (MAD).

The outcomes of the harmonization procedure substantially improve over the original data, with 25% of the data characterized by MAD lower than 0.5 (against 10% of the original GEOV2) and about 90% with MAD < 1 for the harmonized dataset of *z*-scores (vs. 65% for GEOV2). These results are achieved by substantially maintaining most of the original dynamic of the data, since only a simple linear transformation with a rigid time shift is applied. Even if the fraction of the domain with MAD between 0.5 and 1 is still substantial (about 65%), the harmonization procedure achieves a notable reduction of the cells with MAD larger than 1. Moreover, the average correlation between the two anomaly datasets (0.55 ± 0.25) suggests an overall good consistency.

In summary, the bias observed between SPOT-VGT and PROBA-V data is characterized by a small magnitude, that is, however, relevant in case of anomaly studies. This bias can easily be removed by means of a simple linear harmonization applied separately to the two datasets by using the MODIS data as a common benchmark. The harmonization parameters are specific to the input MODIS dataset (after the pre-processing of the original fAPAR MODIS C6 data applied in EDO and GDO as described in section 2.2). It follows that the use of a different fAPAR benchmark dataset may lead to a different parameterization. In addition, it should be point out that this homogenization may alter the magnitude of the original fAPAR time series in an undesirable way, since it focuses on optimizing the computation of temporal consistent anomalies and not on the actual accuracy of fAPAR estimates. Overall, this harmonization procedure provides a bias-corrected GEOV2 dataset that can successfully be used to derive temporally consistent time series of fAPAR anomalies for drought applications.

Further studies will be needed to investigate the actual reliability of near-real time fAPAR anomaly estimates based on the PROBA-V RT0 product rather than the consolidated RT6 dataset tested in this study, as well as to evaluate the future usage of PROBA-V under the effects of a possible degradation of the sensor signal and satellite drifting.
